# The Africa non-communicable diseases (NCD) Open Lab: Impact of a portfolio of clinical studies to deepen the understanding of NCDs in sub-Saharan Africa

**DOI:** 10.7189/jogh.14.04065

**Published:** 2024-05-03

**Authors:** Juliet Addo, Maria Davy, Amy Newlands, Lindsay Orford, Phyllis Guta, Rhona Scott, James van Hasselt, Gareth Maher-Edwards

**Affiliations:** 1 GSK, Brentford, Middlesex, UK; 2 GSK, Stevenage, Hertfordshire, UK; 3 General Medicines Regional Medical Affairs, GSK, Gauteng, South Africa

## Abstract

**Background:**

Clinical research in sub-Saharan Africa (SSA) has often focussed on communicable diseases. However, with the increasing burden of non-communicable diseases (NCDs), there is a need for Africa-specific NCD research.

**Methods:**

GSK established the Africa NCD Open Lab in 2014. Three calls for proposals were advertised through various media channels. An external independent scientific advisory board, predominantly representing African scientists and NCD experts, reviewed and selected projects to receive funding. An additional programme in the Africa NCD Open Lab was designed to build statistical capability by supporting training initiatives. We assessed the impact of the Africa NCD Open Lab in three ways: scientific quality with impact; research training and professional development; and research environments. We captured metrics through regular reports/interactions with researchers; via a final report; and through exit interviews with principal investigators.

**Results:**

Twenty projects in 11 African countries were funded; reports from 18 completed projects are available (data capture is ongoing). Overall, 139 articles have been published in peer-reviewed journals and other data have been presented at conferences and other forums. Most completed projects led to positive outcomes, such as further research, informing policy, or positively impacting clinical care, including three projects that saw changes to regional or national practice guidelines: the CREOLE study in Nigeria; the African Severe Asthma Program in Uganda; and the African Prospective Study on the Early Detection and Identification of Cardiovascular Disease and Hypertension in South Africa. Participation in the Africa NCD Open Lab led to the award of 34 grants related to or influenced by increased research capacity or experience. Significant professional development related to the projects also occurred with higher-level degrees being awarded, including 30 MScs, 30 PhDs, and nine postdoctoral fellowships. Through these projects, research capacity was strengthened across the region by equipping core research facilities, training research staff, strengthening research support services, and supporting the expansion of investigator networks.

**Conclusions:**

The completed Africa NCD Open Lab projects demonstrate high-quality research outcomes addressing important health challenges with potential benefits to African populations. Based on the success of the Africa NCD Open Lab, additional funding has been secured to extend the Open Lab initiative.

Non-communicable diseases (NCDs), such as cardiovascular diseases (CVDs), chronic respiratory diseases, and diabetes, are a major cause of morbidity and mortality worldwide [1]. They disproportionately affect people in low- and middle-income countries, where almost 80% of the total annual global NCD-related deaths (31.4 of 41.0 million) occur [1].

In Africa, more than one-third of annual deaths are due to NCDs, and premature deaths from NCDs in people aged <70 years are rising [2]. NCDs threaten public health and social and economic development in Africa. Furthermore, the dual burden of NCDs and infectious/communicable diseases, such as malaria, tuberculosis, and human immunodeficiency virus (HIV), presents an additional economic and health system strain [3].

Clinical research in sub-Saharan Africa (SSA) is predominantly funded by governments, foundations, or industry, and is often focussed on communicable diseases [4–7]. This leads to research funding and capability gaps in the region, particularly regarding NCDs [8,9]. Furthermore, due to the unique environmental and genetic determinants of NCDs, research findings from high-income countries are not consistently generalisable to Africa, leaving a need for Africa-specific NCD research [10].

In light of these challenges, the GSK Africa NCD Open Lab was established in 2014 with the aim of supporting NCD research in SSA. A key objective was to support African investigators at their local institutions by funding research projects designed to quantify the regional burden of NCDs and to understand unique aspects, such as the drivers and pathophysiology of NCDs, variations in clinical features, and determinants of treatment response. Other key objectives were to directly support the training and research capabilities of African scientists participating in a portfolio of projects, strengthen statistical capability by supporting training initiatives, and to support the development of a new generation of African NCD researchers.

Here we describe the Africa NCD Open Lab, its reach, and its impact and legacy regarding the scientific development and progression of researchers; improvements in infrastructure; and the creation of collaborative networks. We discuss the successes and challenges of the Africa NCD Open Lab, noting gaps and opportunities for future collaborations and partnerships designed to support further research in Africa.

## METHODS

### Call for proposals

Three calls for proposals were launched and advertised through various media channels. Call 1 was launched in 2014 and was aimed at researchers in Cameroon, the Gambia, Ghana, Kenya, Côte d’Ivoire, Malawi, Nigeria, and Uganda [11]. Call 2 was launched in 2015, in collaboration with the South African Medical Research Council and the UK Medical Research Council via the Newton Fund [12], and was open to South African academics and clinicians to reflect the relative regional strength of the country’s research capabilities. Call 3 was launched in 2016 and targeted early-career researchers in Cameroon, the Gambia, Ghana, Kenya, Côte d’Ivoire, Malawi, Nigeria, Uganda, Ethiopia, Senegal, and Tanzania [4].

### Scientific advisory board

An independent scientific advisory board – mainly comprising African scientists – reviewed, short-listed, and selected the project proposals to receive research funding, as detailed further in Gatsi et al. [11] and Addo et al. [4]. In brief, for each project proposal, the scientific advisory board reviewers evaluated the scientific merit; potential significance and impact; the feasibility of the proposed approach; the strategies and methods outlined; the applicant’s research environment and support; and the track record of the investigators [11]. Throughout the scientific advisory board’s review process, any apparent or potential conflicts of interest were disclosed and managed by excluding the conflicted member from the review and discussion on the proposal [11].

### Measuring impact

We assessed the impact of the Africa NCD Open Lab in three ways:

1. Scientific quality with impact: The research objectives and milestones achieved; the number and type of relevant publications and how research findings led to changes (e.g. in local practice; policy and guidelines; and further research and additional grants).
2. Research training and professional development: The number of MSc/PhD qualifications funded; the number and type of sustainable, high-quality training programmes in place; and the extent of improved access to global scientific expertise.
3. Research environments: The physical research infrastructure enabled; the research support functions strengthened; and the networks created and/or facilitated.

Metrics were captured through regular reports and interactions with researchers and via a final report submitted for the projects. We obtained further feedback through exit interviews with principal investigators (PIs) (Appendix S1 in the **Online Supplementary Document**). Information from the exit interviews (collected between 11 June 2020 and 31 May 2023) was collated, analysed, and presented in tables and graphically using bar charts.

## RESULTS

The three calls for proposals resulted in the funding of 20 projects in 11 countries in SSA. Five projects were funded from Call 1 – three in Uganda, and one each in Nigeria and Malawi. They investigated aspects of CVD and interactions between CVD and HIV infection, oncology (breast cancer), chronic respiratory disease (asthma), chronic kidney disease (CKD), and diabetes. Six projects in South Africa were funded from Call 2. They addressed CKD, heart disease, awareness of cancer symptoms in women, the genomics of oesophageal cancer, and determinants of CVD and diabetes. Lastly, nine projects were funded from Call 3 – three in Uganda, two in Tanzania, and one each in Nigeria, Kenya, Malawi, and Ethiopia. They investigated different NCDs and associations with infectious diseases. Therapy areas included diabetes and its complications; chronic obstructive pulmonary disease (COPD); CKD in a birth cohort of children; CVD in pregnancy; and CVD, CKD, and COPD in people living with HIV. To date, 18 out of 20 projects have concluded and have completed exit interviews; the key outcomes of the projects are described in **Table 1** (note that data capture is ongoing).

**Table 1:** Overview of projects funded by the Africa NCD Open Lab compiled based on the exit interviews and in collaboration with the PIs*

Therapy area, PI(s)	Country	Project title	Key project impacts/outcomes	Published references†
**Call 1: Experienced researchers (total GSK investment GBP 4.0 million (USD 5.6 million); typically GBP 0.75–1.0 million (USD 1.05–1.4 million) per project)**				
CVD/CBD, Peterson I & Benjamin L	Malawi	The identification of modifiable viral and inflammatory risk factors for CBD and CVD in HIV-infected African adults	The findings from this study will help identify novel and interacting risk factors, and prioritise CVD and HIV treatment types in a population with a growing CVD burden in SSA.	1
Asthma, Kirenga B & Muttamba W	Uganda, Kenya, Ethiopia	African Severe Asthma Program (ASAP) (ClinicalTrials.gov: NCT03065920)	The national asthma treatment guidelines in Uganda were updated. The essential drugs list for Uganda was updated to include ICSs and ICS/LABA. Model ASAP asthma clinics were established at three study sites and will serve as training centres for health care workers and medical students, as well as promoting patient care.	2–4
CKD, Kalyesubula R	Malawi, Uganda	Characterisation of kidney disease in Malawi and Uganda	This collaborative project demonstrated that CKD prevalence is considerably underestimated in SSA.‡ Existing eGFR equations overestimate GFR in Black African patients; this misclassification cannot be substantially improved by modifying existing equations. These findings have major implications for individual and public health care measures when addressing the challenges posed by renal disease in resource-poor regions.	5–10
Oncology, Niyonzima N & Orem J	Uganda	Defining the molecular profile of breast cancer in Uganda and its clinical implications (ClinicalTrials.gov: NCT03518242)	The study results provide a catalyst to consider the utility of hormone receptor testing more routinely in clinical practice. In addition, the study highlights the potential of less labour-intensive and accessible PCR testing vs the current standard of immunohistochemistry; further research is planned to strengthen data comparing the two techniques.	
CVD, Ojji DB	Nigeria, Cameroon, Kenya, Uganda, Mozambique, South Africa	Comparison of three combination therapies in lowering blood pressure in Black Africans (CREOLE study) (ClinicalTrials.gov: NCT02742467)	In Black patients from SSA, amlodipine + HCTZ and amlodipine + perindopril were significantly more effective than perindopril + HCTZ in lowering blood pressure. Based on these results, the International Society of Hypertension Guidelines (2020) revised the treatment of Black patients with hypertension.	11–17
**Call 2: GBP 5.0 million (USD 6.4 million) collaboration between GSK (20%), the Medical Research Councils of the UK (50%), and South Africa (30%); typically GBP 0.5–1.0 million (USD 0.64–1.28 million) per project**				
Oncology, Moodley J	South Africa, Uganda	Improving timely diagnosis of symptomatic breast and cervical cancer in SSA	Development and validation of the African Women Awareness of CANcer (AWACAN) tool for measurement of breast and cervical cancer awareness. This is the first such tool developed for local use. Mapping community awareness of breast and cervical cancer risk factors, symptoms, lay beliefs, help-seeking behaviour, and barriers to accessing care for potential cancer symptoms in Uganda and South Africa, using the AWACAN tool. Set up the AWACAN network, which is open access and includes participants mainly in Africa (approx. 20 countries to date), and is facilitating collaborations and data sharing relating to early-diagnosis cancer research; the network enables wider access to the AWACAN tool (https://awacan.online).	18–30
CVD, Schutte AE	South Africa	African Prospective Study for the Early Detection and Identification of CVD and Hypertension (African-PREDICT) (ClinicalTrials.gov: NCT03292094)	Metabolomic analyses identified increased collagen biosynthesis in young Black adults, which may suggest a pathway for increased arterial stiffness as a precursor for future hypertension development. The PI was the senior author of the International Society of Hypertension Practice Guidelines (2020); some sections were based on observations from this study. Two biofreezers were purchased to support the long-term biobanking initiative of the study; such biobanks are rare in Africa.	31–85
CVD, Ntusi N & Kraus SM	South Africa, Mozambique	African Cardiomyopathy and Myocarditis Registry Programme: The IMHOTEP Study	This led to the creation of the largest African-based cardiomyopathy cohort to date (>850 patients and >60 families). Two novel genes (CDH2 and POLG) associated with cardiomyopathy were discovered. The predominance of dilated cardiomyopathy and distribution of causes in dilated cardiomyopathy were found to be different from that reported in other countries. Clinical family screening and genetic testing have facilitated the identification of affected family members with asymptomatic disease, resulting in earlier referral to clinical services for treatment. The Inherited Cardiomyopathy Genetics Counselling and Family Screening Initiative was launched by investigators in 2023. As genetic testing for cardiomyopathies is not readily available in southern Africa, this initiative was established to provide feedback on genetic results and counselling to patients with disease-causing mutations identified through the IMHOTEP study. The initiative facilitated the integration of research results into the clinical care record and provided counselling to families.	86–89
Metabolic, Goedecke JH & Micklesfield LK	South Africa	Determinants of T2D and the role of tissue-specific glucocorticoid metabolism and inflammation in middle-aged Black South African men and women (ClinicalTrials.gov: NCT03408678)	This was the first longitudinal study in Africans to determine waist circumference thresholds for predicting dysglycaemia and T2D. The study showed that existing cut-off points for waist circumference were not performing well for Black South Africans, particularly women. Alternative cut-off points applicable to both genders were proposed, with potential to simplify messaging around NCD risk. Sex-specific differences were observed, with Black African men at greater risk for T2D due to lower insulin sensitivity and secretion compared with women. Using targeted proteomics, 73 proteins associated with impaired glucose metabolism and T2D in middle-aged Black Africans were identified, of which 34 were validated in a European cohort and 39 were African-specific. Proteins will be further validated in a longitudinal follow-up of the cohort, potentially improving T2D prediction in Africa. Physical activity and nutrient patterns were associated with diabetes risk and adiposity, with sex-specific associations reported. This was the first study to investigate the interaction between HIV and menopause on bone health in a southern African population. The study showed greater menopause-related bone loss in women with HIV, emphasising the need for routine bone health assessment in middle-aged women with HIV.	90–102
CKD, Naicker S & Fabian J	South Africa	Prevalence, characterisation and response to CKD in South Africa	This collaborative project demonstrated that CKD prevalence is considerably underestimated in SSA. Existing creatinine-based eGFR equations overestimate GFR in Black individuals; this misclassification cannot be substantially improved by modifying existing equations. Cystatin C is a better biomarker for eGFR; however, additional biomarkers are needed in African populations. Formation of the African Research on Kidney Disease Network has allowed a broader network to be formed (https://blogs.lshtm.ac.uk/ark). The point-of-care studies demonstrated the need to make screening for kidney disease accessible, accurate, and affordable.‡	103–112§
Oncology, Parker I	South Africa	A genomic analysis of African oesophageal squamous cell carcinoma in South Africa and Kenya	Our data show that similar molecular pathways are operating in African patients with OSCC compared with European and Asian patients, so targeted therapies developed in HICs may be suitable for African patients with OSCC. Molecular markers from the whole genome/exome sequencing will be useful for early detection of African OSCC via cytosponge or blood plasma samples. We identified 18 initial miRNA candidates and subsequently validated their expression in OSCC tissues. We confirmed the overexpression of 8 miRNAs in serum specimens. Using a serum training cohort, we developed a circulating miRNA signature, and the diagnostic performance of the miRNA signature was confirmed in two independent validation cohorts. Notably, the 8-miRNA signature was superior to current clinical serological markers in distinguishing patients with early-stage OSCC from healthy controls *(P <* .001). Using a novel biomarker discovery approach, we provided the first evidence for a cfDNA methylation assay that offers robust diagnostic accuracy for GI cancers.	113–128
**Call 3: Early career researchers (total GSK funding GBP 1.0 million (USD 1.3 million); typically GBP 0.1 million (USD 0.13 million) per project)**				
CVD, Makubi A & Chillo P	Tanzania	A prospective study of maternal CVDs in Tanzania: Understanding of the aetiological patterns, pathophysiological progression and prognosis	The prevalence of CVD was high among pregnant women (14.6%) and was associated with adverse maternal and foetal outcomes. This study increased the awareness for CVD screening in pregnant women in the institutions and laid a platform for establishing a national/regional maternal CVDs registry in Tanzania and East Africa.	129
CVD, Temu TM	Kenya	Chronic inflammation and early risk of atherosclerosis among HIV-infected adults in Kenya; LTBI is associated with heightened levels of pro- and anti-inflammatory cytokines among Kenyan men and women living with HIV on long-term antiretroviral therapy	Despite viral suppression, patients with HIV had evidence of enhanced endothelial activation associated with sCD14, suggesting that monocyte activation plays a role in atherosclerotic plaque development. Individuals with HIV and LTBI exhibit abnormal cytokine production accompanied by high concentrations of pro- and anti-inflammatory cytokines.	130–134
CVD, Nakimuli A	Uganda	Future maternal cardiovascular health after pre-eclampsia in an indigenous African population	Pre-eclampsia is an independent risk factor for chronic hypertension 1 y post delivery in an indigenous African population. This is one of the first studies to characterise this risk in sub-Saharan women, a population at a very high risk of developing pre-eclampsia and its complications. Women with pre-eclampsia and those with very low education attainment are at increased risk of later hypertension, and these individuals would benefit from prolonged follow-up and early therapeutic intervention. Robust baseline data will allow for investigation of endpoints of CVD after pre-eclampsia. In addition, this study is a platform for more studies on a wider range of pregnancy complications and risk of CVD; the extensive biorepository will also facilitate future genomic analyses.	
Respiratory, Alupo P	Uganda	Frequency and predictors of acute exacerbations of COPD in Uganda	This study demonstrated a high disease burden and exacerbation frequency in patients with COPD in Uganda with unique risk factors compared with patients with COPD in HICs, e.g. HIV and TB. It highlighted the need for more research into effective strategies to prevent and treat COPD in LMICs, such as Uganda, and the need for context-specific guidelines. During the study period, free screening and diagnostic services provided by the study enabled patients who would otherwise not have known about their condition to get diagnosed. A model COPD clinic was established. The study positioned the investigators to advocate for inclusion of COPD in the Uganda clinical guidelines and advocate for inclusion of COPD medications (inhalers) in the Uganda essential medicines list; these have now been included.	
Respiratory, Kayongo A	Uganda	Investigating the role of altered lung microbiome in the pathogenesis of HIV-associated COPD in a Ugandan cohort	Reduced bacterial richness and significant enrichment in *Campylobacter* were associated with HIV-COPD comorbidity. Functional prediction using PICRUSt2 revealed a significant depletion in glutamate degradation capacity pathways in HIV-positive patients. Specific genera (*Staphylococcus* enrichment; *Pseudopropionibacterium* and *Porphyromonas* depletion) were associated with COPD. A comparison of our findings with an HIV cohort from the UK revealed significant differences in the sputum microbiome composition, irrespective of viral suppression. The COPD-related microbiome harbours antimicrobial drug-resistance genes. The study improved access to TB diagnostics in the district. Before the study, there were no facilities available for sputum collection. Since the study, researchers have collected sputum from ≥3 patients per week.	135–137
CKD, Yilma D	Ethiopia	Are kidney function markers associated with low birth weight and stunting in Ethiopian children?	The study provided information on early growth and kidney function in children; a follow-up cohort is needed to assess the subsequent impact in kidney disease. Greater growth between 0 and 6 y of development benefits kidney size; however, greater linear growth velocity after two years is associated with higher serum cystatin C levels. Three PhD students were enrolled for this study, and they received mentorship from various experts through the collaboration in the project. The students were working on investigating early growth factors, cardiometabolic factors and cognitive function, and school performance.	
CKD, Hassan MO	Nigeria	Prevalence and pharmacogenomics of tenofovir nephrotoxicity in HIV-infected adults in South-West Nigeria	The Africa NCD Open Lab grant provided an opportunity to screen and identify patients with HIV on therapy who are at risk of tenofovir-induced nephrotoxicity at HIV treatment centres across South-West Nigeria. Physicians treating these patients were advised to switch the patients to an alternative drug while those patients with significant renal dysfunction are being followed up in a nephrology clinic. A biobank and data repository were established; this will facilitate new collaboration, enabling the study of kidney disease from the perspectives of epidemiology, genetics, and molecular biology. The grant also provided the opportunity to train one MSc student at the University of the Witwatersrand, South Africa, as part of capacity building in nephrology research. The investigator received a fellowship from the Africa Research Excellence Fund, and his research during this fellowship has leveraged the Africa NCD Open Lab cohorts to identify and validate miRNAs that are differentially expressed in patients with HIV who have tenofovir-induced tubulopathy.	138
Metabolic, Chingwanda C	Malawi	Biochemical characterisation of people with diabetes attending the diabetes clinic	The pathophysiological basis of diabetes presentation in Malawians was determined. The study showed that there is often misclassification of patients as T2D when in fact they present with T1D or latent autoimmune diabetes. The study also showed that there is a cohort of patients (23.1%) that do not easily classify as the classic T2D, T1D, or LADA. They present with β-cell failure without autoantibodies at diagnosis. Diabetes management protocols can now be tailored to the individual patient, thus improving treatment response and reducing the prevalence of complications. There is a need to further study the 23.1% of patients to understand the drivers and genetics that result in this presentation.	
Metabolic, Mashili F	Tanzania	Determinants and microbial characterisation of diabetic foot ulcers: Examining factors related to healing and progression of diabetic foot ulcers in an African setting	The study investigated the effect of adiposity and associated microbial factors on healing and progression of DFU in Tanzania. The study showed that impaired lean body mass and low phase angle were significantly associated with delayed healing of DFU. The presence of infection, peripheral arterial disease, and advanced DFU stage were associated with DFU complications, such as amputations and mortality. Overall, this study highlights the importance of body composition and bioimpedance phase angle in DFU healing and progression, as well as the impact of infections, peripheral arterial disease, and advanced DFU stage on DFU complications. These findings can potentially inform clinical management of DFU in Tanzania and other similar settings to improve patient outcomes. The study findings emphasise the importance of a holistic approach to the diagnosis, progress monitoring, and treatment of DFUs. Clinicians should consider not only traditional clinical indicators but also factors related to body composition and bioimpedance phase angle to provide more effective and personalised care for patients with DFU.	139

CBD – cerebrovascular disease, cfDNA – cell-free DNA, CKD – chronic kidney disease, COPD – chronic obstructive pulmonary disease, CVD – cardiovascular disease, DFU – diabetic foot ulcer, eGFR – estimated glomerular filtration rate, GFR – glomerular filtration rate, GI – gastrointestinal, HCTZ – hydrochlorothiazide, HIC – high-income country, HIV – human immunodeficiency virus, ICS – inhaled corticosteroid, LABA – long-acting β-2 agonist, LADA – latent autoimmune diabetes of adults, LMIC – low- and middle-income country, LTBI – latent tuberculosis infection, miRNA – micro RNA, NCD – non-communicable disease, OSCC – oesophageal squamous cell carcinoma, PCR – polymerase chain reaction, PI – principal investigator, PICRUSt2 – Phylogenetic Investigation of Communities by Reconstruction of Unobserved States, sCD14 – soluble CD14, SSA – sub-Saharan Africa, T1D – type 1 diabetes, T2D – type 2 diabetes, TB – tuberculosis

*Brief information is provided about key project outcomes and the impact of the projects completed.

†Articles published in peer-reviewed journals as of 31 May 2023; see the reference list within Appendix S2 in the **Online Supplementary Document** for full citations.

‡The Malawi/Uganda CKD project (first call) and the South African CKD project (second call) were conducted using the same protocol, thus permitting a collaborative approach and pooling of results from three countries in SSA.

§Articles under references 104, 105, 107, and 112 are joint publications across the Malawi/Uganda CKD project (first call) and the South African CKD project (second call).

### Scientific quality with impact

As of 31 May 2023, 139 articles have been published in peer-reviewed journals, including some notable publications in high-impact factor journals, such as *The New England Journal of Medicine* [13], *The Lancet Global Health* [14], *The British Medical Journal* [15], and *The Journal of the American Society of Nephrology* [16] (see full reference list in Appendix S2 in the **Online Supplementary Document**). Further journal publications are expected to follow. Other communications, including conference proceedings, press releases, and reviews have also been outcomes of the Africa NCD Open Lab.

**Figure 1:**
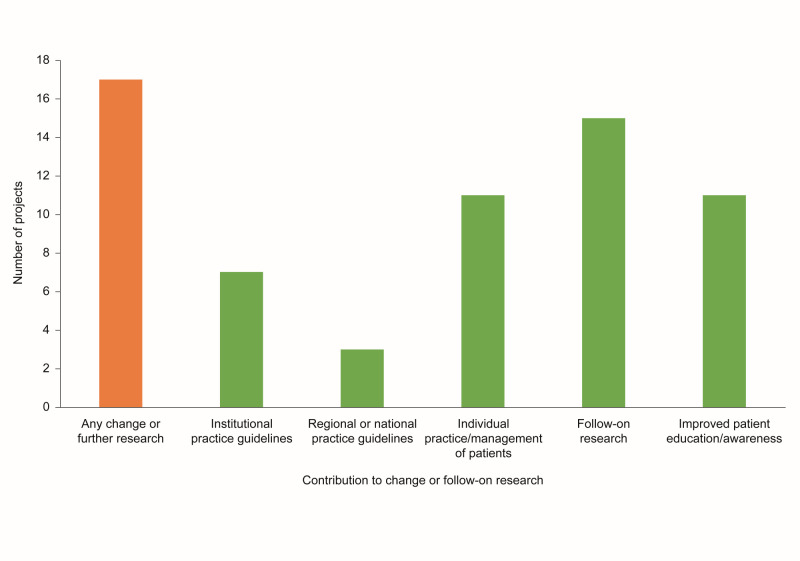
Number of projects in the Africa NCD Open Lab that contributed to change or follow-on research (based on 18 out of 20 exit interviews collected between 11 June 2020 and 31 May 2023). NCD – non-communicable disease.

Most of the completed projects (17 out of 18) contributed to change (e.g. change to institutional, regional, or national guidelines), follow-on research, and improved patient awareness (**Figure 1**). Notably, three projects led to change to regional and national practice guidelines: the CREOLE study in Nigeria [13], the African Severe Asthma Program (ASAP) in Uganda [17], and the African Prospective Study on the Early Detection and Identification of Cardiovascular Disease and Hypertension (African-PREDICT) [18–21].

The CREOLE study showed that the combination of amlodipine with either hydrochlorothiazide or perindopril was superior to perindopril plus hydrochlorothiazide in reducing blood pressure in Black individuals with hypertension in SSA [13]. Previously, guidelines for the treatment of hypertension in Nigeria focussed on combination schedules containing a thiazide diuretic. However, new data from the CREOLE study [13] showed that calcium channel blocker-based combinations are more efficacious than thiazide-based combinations, which will help inform drug choice for hypertension in Black individuals.

The ASAP study led to an update to national asthma treatment guidelines in Uganda, informed in part by some of the project findings [17]. The importance of spirometry in diagnosing and monitoring chronic respiratory disease was recognised by the Ministry of Health and NCD Directorate, and the updated guidelines (yet to be published) will recommend spirometry in all referral and tertiary hospitals and peak flow measurements for those in zonal and district hospitals. The study investigator commented: ‘All of our patients suspected of having airway disease will have spirometry and definitive diagnosis of asthma or COPD will be made and based on guidelines, appropriate management will be started; this helps us to improve the quality of care of our patients and also helps to monitor patients’ response to therapy.’

The African-PREDICT study in South Africa identified distinct ethnic-specific inflammatory mediator patterns and a clearly suppressed renin-angiotensin-aldosterone system profile in Black adults, despite them having similar blood pressures to their White counterparts [18–21]. Metabolomic analyses identified increased collagen biosynthesis in young Black adults, which may suggest a pathway for increased arterial stiffness as a precursor for future hypertension development. Two biofreezers were purchased to support the long-term biobanking initiative of the study. The study’s PI commented: ‘Access to this very unique biobank is likely one of the most valuable assets of the study, with massive potential impact on the long term. Such biobanks are rare in Africa…’ Furthermore, the PI was the senior author of the International Society of Hypertension Practice Guidelines (2020), which included a comprehensive section on ethnicity and lifestyle modifications, some parts of which were based on observations from the African-PREDICT study.

**Figure 2:**
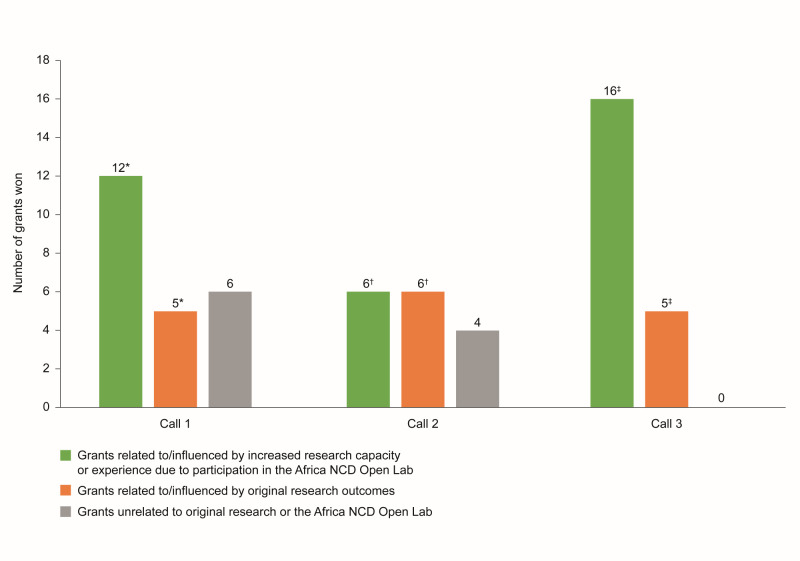
Additional grants won by projects that participated in the Africa NCD Open Lab (based on 18 out of 20 exit interviews collected between 11 June 2020 and 31 May 2023). *Indicates one PI had two grants (one each from research outcomes and capacity building). †Indicates one PI had four grants (three from capacity building (also included in research outcomes), and the other is a faculty research grant). ‡Indicates one PI had four grants, but there is an overlap between the research outcomes and capacity building for all grants. Another PI received an AREF Research Development Fellowship Grant (included under both research outcomes and capacity building). One PI received a World Health Organization/Tropical Disease Research fellowship and a postdoctoral fellowship, and these have both been included along with the grant. For Call 3, grants related to/influenced by original research outcomes are also represented in the column pertaining to grants related to/influenced by increased research capacity or experience due to participation in the Africa NCD Open Lab. AREF – Africa Research Excellence Fund, NCD – non-communicable disease, PI – principal investigator.

Participation in the Africa NCD Open Lab led to the award of 34 external grants related to/influenced by increased research capacity or experience (**Figure 2**). Several investigators reported receipt of awards or recognition. For example, one investigator was awarded a certificate of recognition for a high-impact paper published in the journal *Hypertension* in 2019 [22], while another was awarded runner-up in the best poster competition at a conference, in recognition of outstanding scientific work for an oral and poster presentation [23].

### Research training and professional development

**Figure 3:**
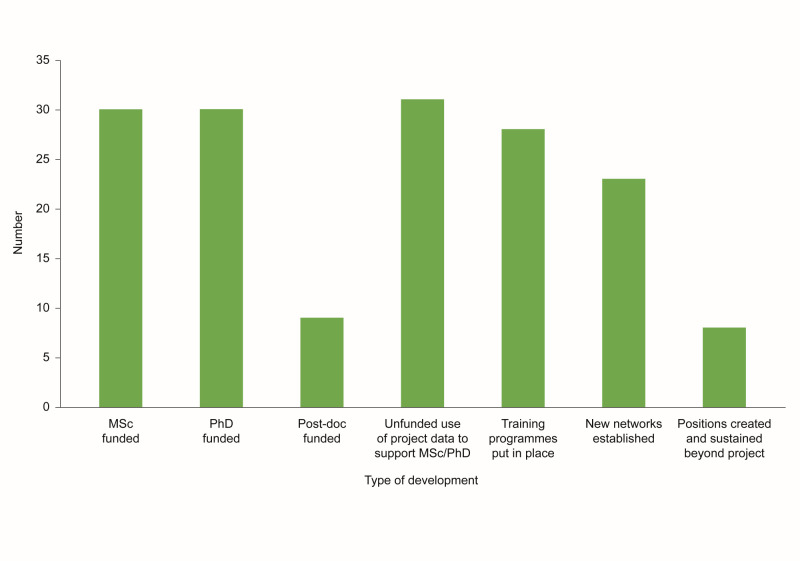
Research training and professional development funded by the Africa NCD Open Lab (based on 18 out of 20 exit interviews collected between 11 June 2020 and 31 May 2023). NCD – non-communicable disease.

The Africa NCD Open Lab resulted in significant professional development opportunities, evidenced by the extent of reported higher-level training, with 30 MScs, 30 PhDs, and nine postdoctoral fellowships being funded (**Figure 3**). One investigator reported that winning a GSK Africa NCD grant strengthened their fellowship application and led to them successfully securing a World Health Organization/Tropical Disease Research fellowship in 2019 and a postdoctoral fellowship at the University of Cape Town in 2021. Furthermore, there was substantial capability training in the Africa NCD Open Lab projects centred around aspects such as Good Clinical Practice, specific additional clinical training, finance, and project management, which was accessible to a broader group than just PIs and students. Capability training across research support groups was key to broader capacity-building impact.

Project participants identified expertise in biomedical statistics as a key need. In response, GSK provided substantial statistical support based on individual project circumstances, including (but not limited to) design discussions and statistical analysis plan input. The Africa NCD Open Lab hosted a one-week, face-to-face statistics summer school in Stevenage, UK, in 2017, to which 18 SSA statisticians supporting projects from the first and second calls were invited. This event enabled the roll-out of technical and non-technical training (e.g. influencing skills and presentation skills) and also helped to build a statistical network among the SSA statisticians, fostering a sense of community and providing a forum for technical problem sharing in the future. Feedback from the investigators was very positive, as seen from the following statements:

- ‘The workshop was very useful and it came at a very important time in my career.’
- ‘The lesson on genetics was very educative and presented in very simple terms to easily understand.’
- ‘I have captured lots of knowledge for future professional application [and] am applying the acquired knowledge at the earliest opportunity.’

Mentorship opportunities were also made possible during the Africa NCD Open Lab, with some of the early career researchers in Call 3 being mentored by the more experienced PIs from Calls 1 and 2. One investigator provided feedback about the benefits they had experienced from being mentored for three years, as well as the wider impact of the project: ‘She [the mentor] has shared most of our findings and helped improve measurements of glomerular filtration rate (GFR). We are trying to work with laboratories to make sure they report the GFR without the risk formula, helping in early diagnosis of patients. This was not being done before. Now we have a big database. There are multiple applications from students across Uganda and other parts of the world, where we can unravel kidney disease in sub-Saharan Africa.’

Driven by the need for experienced and qualified statisticians, a separate, additional programme in the Africa NCD Open Lab aimed to support statistics training initiatives. The Africa NCD Open Lab provided funding for:

- Six PhDs and 10 MScs through the Sub-Saharan Africa Consortium for Advanced Biostatistics training programme, a Wellcome Trust-funded consortium under the Developing Excellence in Leadership, Training and Science in Africa Scheme.
- Three statistics fellowships at The London School of Hygiene and Tropical Medicine (LSHTM). These fellowships ran as two-year blocks and focussed on recruitment from Africa. The first year was an MSc in Medical Statistics at the LSHTM, and the second year was a placement in Africa, with emphasis on NCD-focussed research.
- Six 12-month internships for students who had completed an MSc in Mathematical Sciences at the African Institute for Mathematical Sciences in Tanzania.

### Research environment

Two in-person workshops (in 2018 and 2019, held in Cape Town, South Africa) and one virtual workshop (in 2021, during the coronavirus disease 2019 (COVID-19) pandemic) involving all Africa NCD Open Lab PIs were held to provide networking, mentoring, opportunities to share best practice, and training on topics that would build capabilities, such as scientific (e.g. genomics) and practical skills (e.g. grant writing and publications). An attendee of the workshop commented: ‘I would like to say a huge thank you for a really fantastic, warm, inspiring meeting. It feels we have become a family – and so wonderful to see the growth in all of our projects. It’s hard to sum it all up – but the ripple effects will be far and wide and deep for the African research community… I am deeply appreciative of all the effort made to support us and pull all of us together – it makes us stronger – and together we can achieve so much.’

In addition to the workshops, the PIs could access scientific and statistical expertise from GSK and non-GSK sources throughout the duration of their projects.

Another benefit to the research environment and wider clinical setting beyond the projects is that equipment purchased and used for the research, including ultrasounds and echocardiogram machines, is continuing to support routine clinical services at several sites, where these were previously limited or absent. An example of this is a local rural clinic in Uganda that was equipped with facilities for safe sputum collection in a project recruiting patients with COPD. The equipment and facility are now being used for the collection of sputum samples from patients with tuberculosis (TB), locally providing a safe environment for staff and patients where facilities did not previously exist. Furthermore, in terms of improvements in services, a researcher from one hospital spent some time with another investigator at another site through one of the GSK-funded projects and came back proficient at performing echocardiography. Not only is the researcher now competent at echocardiography and providing thorough reports, which makes a difference in the management of patients, but they have gone on to train further people to enable the service to be established. The echocardiography service is still running despite the researcher having temporarily left to complete training elsewhere. This example highlights how, by training just one person, a valuable service can be made available, not just in one hospital, but also for the rest of the region.

Notably, a ready willingness of the Africa NCD Open Lab researchers to share their expertise maximised the opportunities to extend their research. They demonstrated agility, leadership, and the ability to address various challenges. For example, in a study investigating CKD in South Africa, asymptomatic pyuria was noted in several study participants, indicating a high burden of unrecognised genitourinary pathology. The investigators started screening for schistosomiasis and discovered a significant undiagnosed prevalence in the rural Agincourt community, which was reported to the provincial health authorities [24]. In addition, the Soweto study site offered a dual-energy x-ray absorptiometry (DEXA) scanner to the remote Agincourt research facility, which enabled researchers to study body composition in depth in this rural area. Independent funding was raised to get the DEXA scanner to Agincourt, which was initiated by the study investigators.

### Exit interview feedback

During the exit interviews, we included a free-text field to allow the investigators to comment on their experiences in their own words. One investigator researching CKD in Malawi, Uganda, and South Africa in collaboration with researchers in South Africa commented: *‘*CKD prevalence was significantly underestimated [in SSA]. As a result of the multicentre collaboration anchored in three African countries, replicating the findings across all the sites strengthened the impact of the research results. In addition, the existing equations for estimation of GFR in our study populations are inappropriate and inaccurate, and worsened by including race-based adjustments based on African American ethnicity. The findings confirm that we are underestimating the burden of CKD.*’*

The investigator also highlighted: *‘*… we were unable to model a new GFR estimating equation appropriate for African populations, as creatinine is a poor biomarker for assessing kidney function in our study populations. This has very important implications for the current laboratory standard for CKD diagnosis, highlighting the need to evaluate other biomarkers, such as cystatin C, which performed better than creatinine in our study.*’ *

The initial collaboration referred to by this investigator expanded the networking opportunities for both research groups and led to the formation of the African Research on Kidney Disease (ARK) Network [25].

Another investigator commented that the Africa NCD Open Lab was more than just research development: ‘(There is) an incredible range of people whose lives have been changed and it has widened their horizons for the future.’ Generally, the investigators spoke of gaining skills to transfer knowledge to other colleagues for future research. They also emphasised that the Africa NCD Open Lab had provided researchers with the benefits of collaboration and demonstrated that team achievements can surpass individual efforts. Specifically, one investigator spoke about an awareness of certain conditions to improve patient outcomes, such as the increasing cases of stroke in young people with HIV in Malawi. The investigators also became more aware that TB was implicated as the problem. Consequently, young people with HIV who currently present with stroke are investigated for TB. This study has resulted in a large change in terms of survival of young stroke patients. This is a key example of how a small amount of information from a study has a big impact on the outcome of patients.

## DISCUSSION

NCDs are a major cause of morbidity and mortality in Africa, threatening public health and social and economic development [1,2,26]. In addition, their epidemiology, presentation, management, and prognosis in Africa can substantially differ from those in high-income countries [8]. Understanding and addressing specific factors associated with NCDs in this context can help with identifying justifiable changes and lead to significant benefits in disease management. For example, broader incorporation of standard screening measures (such as blood pressure and peak expiratory flow measurements) into African clinical practice and updating regional and national disease-management guidelines can help SSA countries better respond to the burden of NCDs.

The Africa NCD Open Lab aimed to support NCD research in SSA, quantify the burden of NCDs, and better understand the unique aspects of NCDs in Africa. It is evident from the outcomes presented here that these aims have been met and exceeded in many cases. Not only have many of the projects led to a greater understanding of the NCDs investigated, but the outcomes have been instrumental in leading to changes in the way some NCDs are managed and treated locally, and in some cases, more widely. Furthermore, the training and research capabilities of those participating in these projects has been enhanced, enabling the development of a new generation of African NCD researchers. It is also important to recognise the legacy of the Africa NCD Open Lab in terms of enabling sustainable improvements for the teams and the skills and services they provide, as well as some improvements in infrastructure and equipment. A network of talented African NCD researchers has been established, including individuals with strengthened capabilities in statistics. Crucially, these researchers can transfer knowledge to other projects and colleagues for future research.

Other successes of the Africa NCD Open Lab, which are a testament to the scientific quality of the research and the talent and commitment of the investigators working on the various projects, are evident from the numerous publications produced; project-influenced changes to regional and national guidelines; and researchers gaining recognition and awards for research conducted together with project-facilitated grants for additional research.

Despite the COVID-19 pandemic causing unforeseen challenges to which investigators and researchers had to adapt, considerable effort and diligence by the investigators throughout the pandemic limited the impact. In one project, a significant loss to follow-up occurred due to COVID-19, and in another project, longitudinal data were unavailable (unpublished due to delays from the pandemic). However, in other cases, the pandemic resulted in faster reviews and a new appreciation of the advantages of telemedicine. Another challenge noted by some investigators included the reliance on international laboratories and collaborations for expert sample analysis – this resulted in shipped samples falling to the back of the queue when the pandemic hit, thus delaying the conclusion of some studies. This highlights that further upskilling and improved capacity for these kinds of facilities to be fully operational within SSA could allow investigators to have greater control over their own work and less reliance on competing with other priorities at collaborator facilities. Ambitious timelines were set for all projects including recruitment and follow-up of participants where indicated; as mentioned above, there were delays in achieving these numbers within the timelines for some studies, especially during the pandemic (e.g. lockdown restrictions meant participants were unable to attend the clinics). Regular reports and support with the project management helped minimise these challenges. Other limitations faced during the implementation of the projects included political unrest, strikes by healthcare workers, and challenges with the procurement processes.

## CONCLUSIONS

The Africa NCD Open Lab allowed African scientists to address challenging questions in areas of unmet research need. The findings demonstrate that appropriate infrastructure and support enables African scientists to conduct high-quality research. Relatively small investment has resulted in significant impact on patient care locally through enhanced scientific knowledge, improved skills and services, and the personal development of PIs and their teams, which will enhance local care, as well as future research. In some cases, the impact has been recognised nationally or across borders, including being highlighted as an example of industry best practice in the 2021 Access to Medicines Index [27]. As the Africa NCD Open Lab concludes, focus is shifting towards the sustainability of continuing research in Africa. Further partnerships and research collaborations to support similar grants are needed as demand is high and the current programme has been shown to be highly successful and impactful. Additional funding opportunities are being explored and a new Open Lab initiative focussing on young career scientists working in infectious diseases is currently under way, with projects expected to commence in 2024.

## Additional material


Online Supplementary Document
