# Supplementary material for: The Africa non-communicable diseases (NCD) Open Lab: Impact of a portfolio of clinical studies to deepen the understanding of NCDs in sub-Saharan Africa
Source: J Glob Health. 2024 May 3;14:04065. doi: 10.7189/jogh.14.04065 (PMC11063967; doi:10.7189/jogh.14.04065)
Supplement: Online Supplementary Document [file jogh-14-04065-s001.pdf]

Online Supplementary Documents

Appendix S1: Exit Interview Questionnaire

Africa NCD Open Lab: Impact Exit Interview

**Project No:**	**PI:**	**Date of Interview:**
**Project Title:**		
**Interviewer:**	**Interviewee(s):**	

Scientific Quality with Impact

Were the research objectives achieved? Yes ☐ No ☐ Partial ☐

Were the research milestones achieved? Yes ☐ No ☐ Partial ☐

Comments:

Were the research outcomes communicated? Yes ☐ Please specify below No ☐ *Include planned publications with details of journal to be submitted to and add 'PLANNED' in citation column*

Publications: ☐

Title	Authors	Journal	Impact Factor	Citation (or pending if under review)

Conferences: ☐ *Include future conferences for which abstracts have been accepted. Add details in Conference column if 'other' type of presentation.*

Title	Presenter	Type of Presentation	Conference	National or International	No. Delegates
		NOT COVERED<br>Oral ☐<br>Poster ☐<br>Plenary ☐<br>Other ☐			
		Oral ☐<br>Poster ☐<br>Plenary ☐<br>Other ☐			
		Oral ☐<br>Poster ☐<br>Plenary ☐<br>Other ☐			

Other *(e.g. press release, review article, awards, recognitions)*: ☐

Type of Communication	Presenter / Author	Date	Audience

Have the results of the project contributed to a change?

Begin this interview with an open-ended question, e.g. What was the main impact/outcome of the research?
Bring in the specific categories during the discussion and discuss and capture the consequence of the stated impact.
Ensure clear negative outcomes of the research are captured where they have led to a positive impact.
Probe for and capture reasons why no changes have been observed where this is the case.

	Yes	No	Comments/Details
Change in individual practice/management of patients?	☐	☐	
Change in institutional practice/guidelines?	☐	☐	
Change in regional practice/guidelines?	☐	☐	
Change in National Guidelines?	☐	☐	
Follow-on Research?	☐	☐	
Improved Patient Education/Awareness?	☐	☐	

Other Comments:

Research Training and Professional Development

How has participation in the NCD Open Lab supported the professional development of you and your team?

Number MSc Funded		Number PhD Funded		Unfunded use of project data to support MSc/PhD		Positions created for and sustained beyond NCD Open Lab Project	

Sustainable High-Quality Training Programmes in Place

Training Programme	Number Trained	Impact	Sustainability Plans

Appropriate Access to Scientific Expertise

Was there sufficient access to GSK expertise during the study? Yes ☐ No ☐ If Yes, what was useful? If No, what was missing?

Was there sufficient access to non-GSK expertise during the study? Yes ☐ No ☐ If Yes, what was useful? If No, what was missing?

Research Environments

Physical Research Infrastructure Enabled: Have any new facilities, expertise or equipment been introduced as part of the NCD Open Lab project?

Facilities / Equipment Introduced	Post Study Use	Impact to the Community / Patients

Research Networks Established/Facilitated or Networks for Professional Development Joined (by PI or other team members)

Network	New Network?	Objectives of the Network / What has the Network Facilitated? *(e.g. Learning, Data Sharing, Future Collaborations, Professional Development)*
	☐	
	☐	

Research Support Groups Strengthened:
What support functions (e.g. statistics, finance, project management) have been strengthened as a result of Africa NCD Open Lab participation?

Research Support Group Strengthened	How was this strengthened by NCD Open Lab?	Post-study Impact

Additional Grants won? Yes ☐ No ☐ Enter number of additional grants by study team member below

	Related to / Influenced by Original Research Outcomes	Related to / Influenced by Increased Capacity or Experience as a result of NCD Open Lab Participation	Unrelated to Original Research or NCD Open Lab Participation
Principal Investigator			
Co-Investigator			
Other Research Team Members			
Other Research Teams			

Any Other Feedback not Covered above:

Appendix S2: Publications resulting from the Africa NCD Open Lab (as of 31 May 2023)

1 Peterson I, Ntsui N, Jambo K, Kelly C, Huwa J, Afran L, et al. Evaluating the reactivation of herpesviruses and inflammation as cardiovascular and cerebrovascular risk factors in antiretroviral therapy initiators in an African HIV-infected population (RHICCA): A protocol for a longitudinal cohort study. BMJ Open. 2019;9:e025576. doi:10.1136/bmjopen-2018-025576

2 Kirenga B, Chakaya J, Yimer G, Nyale G, Haile T, Muttamba W, et al. Phenotypic characteristics and asthma severity in an East African cohort of adults and adolescents with asthma: Findings from the African severe asthma project. BMJ Open Respir Res. 2020;7:e000484. Medline:32054641 doi:10.1136/bmjresp-2019-000484

3 Kwizera R, Wadda V, Mugenyi L, Aanyu-Tukamuhebwa H, Nyale G, Yimer G, et al. Skin prick reactivity among asthmatics in East Africa. World Allergy Organ J. 2020;13:100130. Medline:32612738 doi:10.1016/j.waojou.2020.100130

4 Bekel A, Haile T, Mekekasha A, Fuad O, Muttamba W, Mugenyi L, et al. Characterization of asthma and its determinants in Ethiopia: Part of the African Severe Asthma Project. Ethiop Med J. 2021;59:143-51.

5 Gama RM, Kalyesubula R, Fabian J, Mahalingasivam V. NICE takes ethnicity out of estimating kidney function. BMJ. 2021;374:n2159. Medline:34507980 doi:10.1136/bmj.n2159

6 Kalyesubula R, Hau JP, Asiki G, Ssebunya B, Kusemererwa S, Seeley J, et al. Impaired renal function in a rural Ugandan population cohort. Wellcome Open Res. 2018;3:149. Medline:31223661 doi:10.12688/wellcomeopenres.14863.3

7 Nakanga WP, Prynn JE, Banda L, Kalyesubula R, Tomlinson LA, Nyirenda M, et al. Prevalence of impaired renal function among rural and urban populations: Findings of a cross-sectional study in Malawi. Wellcome Open Res. 2019;4:92. Medline:31656860 doi:10.12688/wellcomeopenres.15255.1

8 Seeley J, Kabunga E, Ssembatya J, Tomlinson LA, Fabian J, Smeeth L, et al. Understanding kidney disease in rural central Uganda - Findings from a qualitative study. Glob Public Health. 2020;15:1566-77. Medline:32352888 doi:10.1080/17441692.2020.1758186

9 Kalyesubula R, Sekitoleko I, Tomlin K, Hansen CH, Ssebunya B, Makanga R, et al. Association of impaired kidney function with mortality in rural Uganda: Results of a general population cohort study. BMJ Open. 2022;12:e051267. Medline:35473721 doi:10.1136/bmjopen-2021-051267

10 Ingenhoff R, Brewster U, Rastegar A, Kalyesubula R, Knauf F. Global health education in nephrology: The time has come. J Am Soc Nephrol. 2021;32:2990-3. Medline:34645697 doi:10.1681/asn.2021060731

11 Ingabire PM, Ojji DB, Rayner B, Ogola E, Damasceno A, Jones E, et al. High prevalence of non-dipping patterns among Black Africans with uncontrolled hypertension: A secondary analysis of the CREOLE trial. BMC Cardiovasc Disord. 2021;21:254. doi:10.1186/s12872-021-02074-7

12 Ojji DB, Cornelius V, Partington G, Francis V, Pandie S, Smythe W, et al. Effect of 3, 2-drug combinations of antihypertensive therapies on blood pressure variability in Black African patients: Secondary analyses of the CREOLE trial. Hypertension. 2022;79:2593-600. Medline:36052684 doi:10.1161/hypertensionaha.121.18333

13 Ojji DB, Mayosi B, Francis V, Badri M, Cornelius V, Smythe W, et al. Comparison of dual therapies for lowering blood pressure in Black Africans. N Engl J Med. 2019;380:2429-39. Medline:30883050 doi:10.1056/NEJMoa1901113

14 Ojji DB, Poulter N, Damasceno A, Sliwa K, Smythe W, Kramer N, et al. Rationale and design of the comparison of 3 combination therapies in lowering blood pressure in Black Africans (CREOLE study): 2 × 3 factorial randomized single-blind multicenter trial. Am Heart J. 2018;202:5-12. Medline:29800784 doi:10.1016/j.ahj.2018.03.023

15 Ojji DB, Shedul GL, Sani M, Ogah OS, Dzudie A, Barasa F, et al. A Differential response to antihypertensive therapy in African men and women: Insights from the CREOLE trial. Am J Hypertens. 2022;35:551-60. Medline:35134817 doi:10.1093/ajh/hpac014

16 Faconti L, McNally RJ, Farukh B, Adeyemi O, Cruickshank JK, Wilkinson IB, et al. Differences in hypertension phenotypes between Africans and Europeans: Role of environment. J Hypertens. 2020;38:1278-85. Medline:32205559 doi:10.1097/hjh.0000000000002403

17 Ojji DB, Poulter N. Comparison of dual therapies for lowering blood pressure in Black Africans. Reply. N Engl J Med. 2019;381:1392. Medline:31577888 doi:10.1056/NEJMc1909844

18 Moodley J, Walter FM, Scott SE, Mwaka AM. Towards timely diagnosis of symptomatic breast and cervical cancer in South Africa. S Afr Med J. 2018;108:803-4. Medline:30421705 doi:10.7196/SAMJ.2018.v108i10.13478

19 Githaiga JN, Walter FM, Scott SE, Mwaka AD, Moodley J. Symptom awareness measures for breast and cervical cancer in sub-Saharan Africa: A scoping review. SAJO. 2019;3:1-9. doi:10.4102/sajo.v3i0.78

20 Moodley J, Scott SE, Mwaka AD, Constant D, Githaiga JN, Stewart TS, et al. Development and validation of the African Women Awareness of CANcer (AWACAN) tool for breast and cervical cancer. PLoS One. 2019;14:e0220545. Medline:31386684 doi:10.1371/journal.pone.0220545

21 Nnaji CA, Kuodi P, Walter FM, Moodley J. Effectiveness of interventions for improving timely diagnosis of breast and cervical cancers in low and middle-income countries: A systematic review protocol. BMJ Open. 2020;10:e042788. doi:10.1136/bmjopen-2020-042788

22 Adoch W, Garimoi CO, Scott SE, Okeny GG, Moodley J, Komakech H, et al. Knowledge of cervical cancer risk factors and symptoms among women in a refugee settlement: A cross-sectional study in northern Uganda. Confl Health. 2020;14:85. doi:10.1186/s13031-020-00328-3

23 Harries J, Scott SE, Walter FM, Mwaka AD, Moodley J. Women's appraisal, interpretation and help-seeking for possible symptoms of breast and cervical cancer in South Africa: A qualitative study. BMC Womens Health. 2020;20:251. Medline:33187501 doi:10.1186/s12905-020-01120-4

24 Moodley J, Constant D, Mwaka AD, Scott SE, Walter FM. Mapping awareness of breast and cervical cancer risk factors, symptoms and lay beliefs in Uganda and South Africa. PLoS One. 2020;15:e0240788. Medline:33091035 doi:10.1371/journal.pone.0240788

25 Moodley J, Constant D, Mwaka AD, Scott SE, Walter FM. Anticipated help seeking behaviour and barriers to seeking care for possible breast and cervical cancer symptoms in Uganda and South Africa. Ecancermedicalscience. 2021;15:1171. Medline:33680085 doi:10.3332/ecancer.2021.1171

26 Moodley J, Harries J, Scott SE, Mwaka AD, Saji S, Walter FM. Exploring primary care level provider interpretation and management of potential breast and cervical cancer signs and symptoms in South Africa. Ecancermedicalscience. 2021;15:1298. Medline:34824621 doi:10.3332/ecancer.2021.1298

27 Nnaji CA, Kuodi P, Walter FM, Moodley J. Timeliness of diagnosis of breast and cervical cancers and associated factors in low-income and middle-income countries: A scoping review protocol. BMJ Open. 2021;11:e044093. Medline:33958339 doi:10.1136/bmjopen-2020-044093

28 Mwaka AD, Walter FM, Scott S, Harries J, Wabinga H, Moodley J. Symptom appraisal, help-seeking and perceived barriers to healthcare seeking in Uganda: An exploratory study among women with potential symptoms of breast and cervical cancer. BMJ Open. 2021;11:e041365. Medline:33550241 doi:10.1136/bmjopen-2020-041365

29 Nnaji CA, Ezenwankwo EF, Kuodi P, Walter FM, Moodley J. Timeliness of diagnosis of breast and cervical cancers and associated factors in low-income and middle-income countries: A scoping review. BMJ Open. 2022;12:e057685. Medline:35121607 doi:10.1136/bmjopen-2021-057685

30 Nnaji CA, Kuodi P, Walter FM, Moodley J. Effectiveness of interventions for improving timely diagnosis of breast and cervical cancers in low-income and middle-income countries: A systematic review. BMJ Open. 2022;12:e054501. Medline:35470184 doi:10.1136/bmjopen-2021-054501

31 Ahiante BO, Smith W, Lammertyn L, Schutte AE. Leptin and the retinal microvasculature in young Black and White adults: The African-PREDICT study. Heart Lung Circ. 2020;29:1823-31. Medline:32622912 doi:10.1016/j.hlc.2020.05.093

32 Ahiante BO, Smith W, Lammertyn L, Schutte AE. Leptin and its relation to autonomic activity, endothelial cell activation and blood pressure in a young Black and White population: The African-PREDICT study. Horm Metab Res. 2018;50:257-66. Medline:29179226 doi:10.1055/s-0043-122236

33 Ahiante BO, Smith W, Lammertyn L, Schutte AE. Leptin and the vasculature in young adults: The African-PREDICT study. Eur J Clin Invest. 2019;49:e13039. Medline:30347447 doi:10.1111/eci.13039

34 Barnard SA, Smith W, Mels CMC, Botha S, Schutte AE. Bioavailable IGF-1 is beneficially associated with biomarkers of endothelial function in young healthy adults: The African-PREDICT study. Growth Horm IGF Res. 2018;41:28-33. Medline:29936324 doi:10.1016/j.ghir.2018.06.002

35 Bollenbach A, Schutte AE, Kruger R, Tsikas D. An ethnic comparison of arginine dimethylation and cardiometabolic factors in healthy Black and White youth: The ASOS and African-PREDICT studies. J Clin Med. 2020;9:844. Medline:32244968 doi:10.3390/jcm9030844

36 Botha D, Breet Y, Schutte AE. Comparing the associations of clinic vs. ambulatory blood pressure with subclinical organ damage in young healthy adults: The African-PREDICT study. Hypertens Res. 2021;44:840-9. Medline:33564179 doi:10.1038/s41440-021-00627-z

37 Breet Y, Huisman HW, Kruger R, van Rooyen JM, Gafane-Matemane LF, Ware LJ, et al. Pulse pressure amplification and its relationship with age in young, apparently healthy black and white adults: The African-PREDICT study. Int J Cardiol. 2017;249:387-91. Medline:28893431 doi:10.1016/j.ijcard.2017.08.070

38 Craig A, Mels CMC, Tsikas D, Boeger RH, Schwedhelm E, Schutte AE, et al. Central systolic blood pressure relates inversely to nitric oxide synthesis in young black adults: The African-PREDICT study. J Hum Hypertens. 2021;35:985-93. Medline:33273698 doi:10.1038/s41371-020-00453-9

39 Craig A, Mels CMC, Schutte AE, Bollenbach A, Tsikas D, Schwedhelm E, et al. Urinary albumin-to-creatinine ratio is inversely related to nitric oxide synthesis in young black adults: The African-PREDICT study. Hypertens Res. 2021;44:71-9. Medline:32681162 doi:10.1038/s41440-020-0514-1

40 Craig A, Mels CMC, Schutte AE, Tsikas D, Kruger R. Nitric oxide-related markers link inversely to blood pressure in black boys and men: The ASOS and African-PREDICT studies. Amino Acids. 2020;52:639-48. Medline:32303905 doi:10.1007/s00726-020-02842-3

41 Crouch SH, Botha-Le Roux S, Delles C, Graham LA, Schutte AE. Inflammation and hypertension development: A longitudinal analysis of the African-PREDICT study. Int J Cardiol Hypertens. 2020;7:100067. Medline:33392493 doi:10.1016/j.ijchy.2020.100067

42 Crouch SH, Botha-Le Roux S, Delles C, Graham LA, Schutte AE. Inflammation and salt in young adults: The African-PREDICT study. Eur J Nutr. 2021;60:873-82. Medline:32494865 doi:10.1007/s00394-020-02292-3

43 Crouch SH, Roux SB, Delles C, Graham LA, Schutte AE. Distinct inflammatory mediator patterns in young black and white adults: The African-predict study. Cytokine. 2020;126:154894. Medline:31670005 doi:10.1016/j.cyto.2019.154894

44 Crouch SH, Ware LJ, Gafane-Matemane LF, Kruger HS, Van Zyl T, Van der Westhuizen B, et al. Dietary sodium intake and its relationship to adiposity in young black and white adults: The African-PREDICT study. J Clin Hypertens (Greenwich). 2018;20:1193-202. Medline:29961983 doi:10.1111/jch.13329

45 De Beer D, Mels CM, Schutte AE, Louw R, Delles C, Kruger R. Left ventricular mass and urinary metabolomics in young black and white adults: The African-PREDICT study. Nutr Metab Cardiovasc Dis. 2020;30:2051-62. Medline:32669241 doi:10.1016/j.numecd.2020.06.004

46 de Beer D, Mels CMC, Schutte AE, Delles C, Mary S, Mullen W, et al. A urinary peptidomics approach for early stages of cardiovascular disease risk: The African-PREDICT study. Hypertens Res. 2023;46:485-94. Medline:36396816 doi:10.1038/s41440-022-01097-7

47 du Toit WL, Kruger R, Gafane-Matemane LF, Schutte AE, Louw R, Mels CMC. Urinary metabolomics profiling by cardiovascular risk factors in young adults: The African prospective study on early detection and identification of cardiovascular disease and hypertension study. J Hypertens. 2022;40:1545-55. Medline:35788095 doi:10.1097/hjh.0000000000003182

48 du Toit WL, Schutte AE, Gafane-Matemane LF, Kruger R, Mels CMC. The renin-angiotensin-system and left ventricular mass in young adults: The African-PREDICT study. Blood Press. 2021;30:98-107. Medline:33084438 doi:10.1080/08037051.2020.1831902

49 Du Toit WL, Schutte AE, Mels CMC. The relationship of blood pressure with uric acid and bilirubin in young lean and overweight/obese men and women: The African-PREDICT study. J Hum Hypertens. 2020;34:648-56. Medline:31712713 doi:10.1038/s41371-019-0287-7

50 Gafane-Matemane LF, Kruger R, Smith W, Mels CMC, Van Rooyen JM, Mokwatsi GG, et al. Characterization of the renin-angiotensin-aldosterone system in young healthy Black adults: The African prospective study on the early detection and identification of hypertension and cardiovascular disease (African-PREDICT study). Hypertension. 2021;78:400-10. Medline:34176281 doi:10.1161/hypertensionaha.120.16879

51 Gafane-Matemane LF, Kruger R, Van Rooyen JM, Gona PN, Schutte AE. Soluble (pro)renin receptor is adversely associated with indices of left ventricular structure and function: The African-PREDICT study. J Cardiovasc Dev Dis. 2022;9:130. Medline:35621841 doi:10.3390/jcdd9050130

52 Gafane-Matemane LF, Mokae NL, Breet Y, Poglitsch M, Schutte AE. Associations of central and peripheral blood pressure with the renin-angiotensin-aldosterone system in healthy young adults: The African-PREDICT study. Hypertens Res. 2021;44:435-45. doi:10.1038/s41440-020-00566-1

53 Jacobs A, Pieters M, Schutte AE. The association of PAI-1 with 24 h blood pressure in young healthy adults is influenced by smoking and alcohol use: The African-PREDICT study. Nutr Metab Cardiovasc Dis. 2020;30:2063-71. Medline:32811735 doi:10.1016/j.numecd.2020.07.003

54 Jacobs A, Pieters M, Smith W, Schutte AE. Retinal vasodilatory responses are inversely associated with plasminogen activator inhibitor-1: The African-PREDICT study. Microvasc Res. 2021;137:104180. Medline:34015274 doi:10.1016/j.mvr.2021.104180

55 Köchli S, Schutte AE, Botha-Le Roux S, Gafane-Matemane LF, Smith W, van Rooyen JM, et al. Potassium excretion and blood pressure are associated with heart rate variability in healthy black adults: The African-PREDICT study. Nutr Metab Cardiovasc Dis. 2021;31:1071-80. Medline:33549447 doi:10.1016/j.numecd.2020.12.021

56 Köchli S, Schutte AE, Kruger R. Adiposity and physical activity are related to heart rate variability: The African-PREDICT study. Eur J Clin Invest. 2020;50:e13330. Medline:32589287 doi:10.1111/eci.13330

57 Kriel JI, Fourie CM, Schutte AE. Monocyte chemoattractant protein-1 and large artery structure and function in young individuals: The African-PREDICT study. J Clin Hypertens (Greenwich). 2017;19:67-74. Medline:27453537 doi:10.1111/jch.12868

58 Mokwatsi GG, Schutte AE, Mels CMC, Kruger R. Morning blood pressure surge in young black and white adults: The African-PREDICT Study. J Hum Hypertens. 2019;33:22-33. Medline:30038337 doi:10.1038/s41371-018-0089-3

59 Möller C, Schutte AE, Smith W, Botha-Le Roux S. Von Willebrand factor, its cleaving protease (ADAMTS13), and inflammation in young adults: The African-PREDICT study. Cytokine. 2020;136:155265. Medline:32927287 doi:10.1016/j.cyto.2020.155265

60 Navise NH, Lammertyn L, Mokwatsi GG, Schutte AE. The association of von willebrand factor and its cleaving protease (ADAMTS13) with health behaviours in young black and white adults: The African-PREDICT study. Biomarkers. 2021;26:508-16. Medline:33938341 doi:10.1080/1354750x.2021.1923063

61 Ramoshaba NE, Huisman HW, Lammertyn L, Kotliar KE, Schutte AE, Smith W. Retinal microvasculature and masked hypertension in young adults: The African-PREDICT study. Hypertens Res. 2020;43:1231-8. doi:10.1038/s41440-020-0487-0

62 Maritz M, Fourie CM, Van Rooyen JM, Moss SJ, Schutte AE. Large artery stiffness is associated with gamma-glutamyltransferase in young, healthy adults: The African-PREDICT study. J Am Soc Hypertens. 2016;10:772-81.e1. Medline:27613367 doi:10.1016/j.jash.2016.07.006

63 Maritz M, Fourie CMT, Van Rooyen JM, Schutte AE. Evaluating several biomarkers as predictors of aortic stiffness in young and older Africans, not consuming alcohol based on self-report. Diabetes Res Clin Pract. 2018;142:312-20. Medline:29906479 doi:10.1016/j.diabres.2018.05.048

64 Maugana VF, Kruger R, Schutte AE, Smith W. Socioeconomic inequalities, modifiable lifestyle risk factors, and retinal vessel calibers: The African-PREDICT study. Microcirculation. 2021;28:e12714. Medline:34008905 doi:10.1111/micc.12714

65 Mels CM, Delles C, Louw R, Schutte AE. Central systolic pressure and a nonessential amino acid metabolomics profile: The African prospective study on the early detection and identification of cardiovascular disease and hypertension. J Hypertens. 2019;37:1157-66. Medline:30801385 doi:10.1097/hjh.0000000000002040

66 Mokwatsi GG, Schutte AE, Mels CMC, Kruger R. Morning blood pressure surge relates to autonomic neural activity in young non-dipping adults: The African-PREDICT study. Heart Lung Circ. 2019;28:1197-205. Medline:30093314 doi:10.1016/j.hlc.2018.07.003

67 Schutte AE, Gona PN, Delles C, Uys AS, Burger A, Mels CM, et al. The African prospective study on the early detection and identification of cardiovascular disease and hypertension (African-PREDICT): Design, recruitment and initial examination. Eur J Prev Cardiol. 2019;26:458-70. Medline:30681377 doi:10.1177/2047487318822354

68 Sekoba NP, Kruger R, Labuschagne P, Schutte AE. Left ventricular mass independently associates with masked hypertension in young healthy adults: The African-PREDICT study. J Hypertens. 2018;36:1689-96. Medline:29601412 doi:10.1097/hjh.0000000000001740

69 Smith W, Kotliar KE, Lammertyn L, Ramoshaba NE, Vilser W, Huisman HW, et al. Retinal vessel caliber and caliber responses in true normotensive black and white adults: The African-PREDICT study. Microvasc Res. 2020;128:103937. Medline:31644892 doi:10.1016/j.mvr.2019.103937

70 Strauss M, Smith W, Kruger R, van der Westhuizen B, Schutte AE. Large artery stiffness is associated with salt intake in young healthy black but not white adults: The African-PREDICT study. Eur J Nutr. 2018;57:2649-56. Medline:30032457 doi:10.1007/s00394-018-1791-1

71 Strauss M, Smith W, Kruger R, Wei W, Fedorova OV, Schutte AE. Marinobufagenin and left ventricular mass in young adults: The African-PREDICT study. Eur J Prev Cardiol. 2018;25:1587-95. Medline:29993282 doi:10.1177/2047487318788140

72 Strauss M, Smith W, Schutte AE. Inter-arm blood pressure difference and its relationship with retinal microvascular calibres in young individuals: The African-PREDICT study. Heart Lung Circ. 2016;25:855-61. Medline:27012863 doi:10.1016/j.hlc.2016.01.019

73 Strauss M, Smith W, Wei W, Bagrov AY, Fedorova OV, Schutte AE. Large artery stiffness is associated with marinobufagenin in young adults: The African-PREDICT study. J Hypertens. 2018;36:2333-9. Medline:30382957 doi:10.1097/hjh.0000000000001866

74 Strauss M, Smith W, Wei W, Fedorova OV, Schutte AE. Autonomic activity and its relationship with the endogenous cardiotonic steroid marinobufagenin: The African-PREDICT study. Nutr Neurosci. 2020;23:849-59. Medline:30614779 doi:10.1080/1028415x.2018.1564985

75 Strauss M, Smith W, Wei W, Fedorova OV, Schutte AE. Marinobufagenin is related to elevated central and 24-h systolic blood pressures in young black women: The African-PREDICT Study. Hypertens Res. 2018;41:183-92. Medline:29335615 doi:10.1038/s41440-017-0009-x

76 Strauss-Kruger M, Kruger R, Smith W, Gafane-Matemane LF, Mokwatsi G, Wei W, et al. The cardiotonic steroid marinobufagenin is a predictor of increased left ventricular mass in obesity: The African-PREDICT study. Nutrients. 2020;12:3185. Medline:33081045 doi:10.3390/nu12103185

77 Strauss-Kruger M, Smith W, Wei W, Bagrov AY, Fedorova OV, Schutte AE. Microvascular function in non-dippers: Potential involvement of the salt sensitivity biomarker, marinobufagenin-The African-PREDICT study. J Clin Hypertens (Greenwich). 2020;22:86-94. Medline:31873989 doi:10.1111/jch.13767

78 Strauss-Kruger M, van Zyl T, Pieters M, Kruger R, Mokwatsi G, Gafane-Matemane L, et al. Urinary metabolomics, dietary salt intake and blood pressure: The African-PREDICT study. Hypertens Res. 2023;46:175-86. Medline:36229536 doi:10.1038/s41440-022-01071-3

79 Strauss-Kruger M, Wentzel-Viljoen E, Ware LJ, Van Zyl T, Charlton K, Ellis S, et al. Early evidence for the effectiveness of South Africa's legislation on salt restriction in foods: The African-PREDICT study. J Hum Hypertens. 2023;37:42-9. Medline:35091704 doi:10.1038/s41371-021-00653-x

80 Swart R, Schutte AE, van Rooyen JM, Smith W, Mels CMC. The association of measures of the micro- and macro-vasculature with selenium and GPx activity in a young bi-ethnic population: The African-PREDICT study. J Am Coll Nutr. 2019;38:614-22. Medline:30822227 doi:10.1080/07315724.2019.1570479

81 Thompson JE, Smith W, Ware LJ, Mels CMC, van Rooyen JM, Huisman HW, et al. Masked hypertension and its associated cardiovascular risk in young individuals: The African-PREDICT study. Hypertens Res. 2016;39:158-65. Medline:26606873 doi:10.1038/hr.2015.123

82 van der Westhuizen B, Schutte AE, Gafane-Matemane LF, Kruger R. Left ventricular mass independently associates with 24-hour sodium excretion in young masked hypertensive adults: The African-PREDICT study. Int J Cardiol. 2019;276:218-23. Medline:30503186 doi:10.1016/j.ijcard.2018.11.116

83 van Rooyen JM, Poglitsch M, Mels CMC, Huisman HW, Gafane-Matemane LF, Le Roux S, et al. Aldosterone and angiotensin II profiles in young black and white women using different hormonal contraceptives: The African-PREDICT study. J Hum Hypertens. 2022;36:711-7. Medline:34172825 doi:10.1038/s41371-021-00569-6

84 Vermeulen B, Gafane-Matemane LF, Schutte AE, Kruger R. Growth differentiating factor-15 and adiposity in young adults: The African-PREDICT study. Int J Obes (Lond). 2021;45:1418-27. Medline:33753886 doi:10.1038/s41366-021-00803-8

85 Vermeulen B, Schutte AE, Gafane-Matemane LF, Kruger R. Growth differentiating factor-15 and its association with traditional cardiovascular risk factors: The African-PREDICT study. Nutr Metab Cardiovasc Dis. 2020;30:925-31. Medline:32278609 doi:10.1016/j.numecd.2020.03.001

86 Kraus SM, Shaboodien G, Francis V, Laing N, Cirota J, Chin A, et al. Rationale and design of the African cardiomyopathy and myocarditis registry program: The IMHOTEP study. Int J Cardiol. 2021;333:119-26. Medline:33607192 doi:10.1016/j.ijcard.2021.02.026

87 Spracklen TF, Kasher PR, Kraus S, Botha TL, Page DJ, Kamuli S, et al. Identification of a POLG variant in a family with arrhythmogenic cardiomyopathy and left ventricular fibrosis. Circ Genom Precis Med. 2021;14:e003138. Medline:33276707 doi:10.1161/circgen.120.003138

88 Mayosi BM, Fish M, Shaboodien G, Mastantuono E, Kraus S, Wieland T, et al. Identification of cadherin 2 (CDH2) mutations in arrhythmogenic right ventricular cardiomyopathy. Circ Cardiovasc Genet. 2017;10:e001605. Medline:28280076 doi:10.1161/circgenetics.116.001605

89 Fish M, Shaboodien G, Kraus S, Sliwa K, Seidman CE, Burke MA, et al. Mutation analysis of the phospholamban gene in 315 South Africans with dilated, hypertrophic, peripartum and arrhythmogenic right ventricular cardiomyopathies. Sci Rep. 2016;6:22235. doi:10.1038/srep22235

90 Dlamini SN, Choudhury A, Ramsay M, Micklesfield LK, Norris SA, Crowther NJ, et al. Associations between CYP17A1 and SERPINA6/A1 polymorphisms, and cardiometabolic risk factors in Black South Africans. Front Genet. 2021;12:687335. Medline:34484290 doi:10.3389/fgene.2021.687335

91 Dlamini SN, Lombard Z, Micklesfield LK, Crowther N, Norris SA, Snyman T, et al. Glucocorticoids associate with cardiometabolic risk factors in Black South Africans. Endocr Connect. 2021;10:873-84. Medline:34261039 doi:10.1530/ec-21-0195

92 Dlamini SN, Norris SA, Mendham AE, Mtintsilana A, Ward KA, Olsson T, et al. Targeted proteomics of appendicular skeletal muscle mass and handgrip strength in black South Africans: A cross-sectional study. Sci Rep. 2022;12:9512. doi:10.1038/s41598-022-13548-9

93 Goedecke JH, Nguyen KA, Kufe C, Masemola M, Chikowore T, Mendham AE, et al. Waist circumference thresholds predicting incident dysglycaemia and type 2 diabetes in Black African men and women. Diabetes Obes Metab. 2022;24:918-27. Medline:35088498 doi:10.1111/dom.14655

94 Kufe CN, Goedecke JH, Masemola M, Chikowore T, Soboyisi M, Smith A, et al. Physical behaviors and their association with type 2 diabetes mellitus risk markers in urban South African middle-aged adults: An isotemporal substitutionapproach. BMJ Open Diabetes Res Care. 2022;10 doi:10.1136/bmjdrc-2022-002815

95 Kufe CN, Micklesfield LK, Masemola M, Chikowore T, Kengne AP, Karpe F, et al. Increased risk for type 2 diabetes in relation to adiposity in middle-aged Black South African men compared to women. Eur J Endocrinol. 2022;186:523-33. Medline:35225824 doi:10.1530/eje-21-0527

96 Kufe NC, Masemola M, Chikowore T, Kengne AP, Olsson T, Goedecke JH, et al. Protocol for systematic review and meta-analysis of sex hormones and diabetes risk in ageing men and women of African ancestry. BMJ Open. 2019;9:e024446. Medline:30610025 doi:10.1136/bmjopen-2018-024446

97 Madanhire T, Goedecke JH, Ward KA, Jaff N, Crowther NJ, Norris S, et al. The impact of human immunodeficiency virus and menopause on bone mineral density: A longitudinal study of urban-dwelling South African women. J Bone Miner Res. 2023;38:619-30. Medline:36726211 doi:10.1002/jbmr.4765

98 Makura-Kankwende CBT, Gradidge PJ, Crowther NJ, Ratshikombo T, Goedecke JH, Micklesfield LK, et al. Association of longitudinal nutrient patterns with body composition in Black middle-aged South African Women: A five-year follow-up study. Int J Environ Res Public Health. 2022;19 Medline:36232088 doi:10.3390/ijerph191912792

99 Mendham AE, Goedecke JH, Kufe NC, Soboyisi M, Smith A, Westgate K, et al. Physical behaviors and their association with adiposity in men and women from a low-resourced African setting. J Phys Act Health. 2022;19:548-57. Medline:35894904 doi:10.1123/jpah.2022-0032

100 Mendham AE, Micklesfield LK, Karpe F, Kengne AP, Chikowore T, Kufe CN, et al. Targeted proteomics identifies potential biomarkers of dysglycaemia, beta cell function and insulin sensitivity in Black African men and women. Diabetologia. 2023;66:174-89. Medline:36114877 doi:10.1007/s00125-022-05788-1

101 Micklesfield LK, Westgate K, Smith A, Kufe CN, Mendham AE, Lindsay T, et al. Physical activity behaviors of a middle-age South African cohort as determined by integrated hip and thigh accelerometry. Med Sci Sports Exerc. 2022;54:1493-505. Medline:35658390 doi:10.1249/mss.0000000000002940

102 Ratshikombo T, Goedecke JH, Soboyisi M, Kufe C, Makura-Kankwende CBT, Masemola M, et al. Sex differences in the associations of nutrient patterns with total and regional adiposity: A study of middle-aged Black South African men and women. Nutrients. 2021;13:4558. Medline:34960108 doi:10.3390/nu13124558

103 Naidoo S, Kagura J, Fabian J, Norris SA. Early life factors and longitudinal blood pressure trajectories are associated with elevated blood pressure in early adulthood. Hypertension. 2019;73:301-9. Medline:30580689 doi:10.1161/hypertensionaha.118.11992

104 Fabian J, George JA, Etheredge HR, van Deventer M, Kalyesubula R, Wade AN, et al. Methods and reporting of kidney function: A systematic review of studies from sub-Saharan Africa. Clin Kidney J. 2019;12:778-87. doi:10.1093/ckj/sfz089

105 Kalyesubula R, Fabian J, Nakanga W, Newton R, Ssebunnya B, Prynn J, et al. How to estimate glomerular filtration rate in sub-Saharan Africa: Design and methods of the African Research into Kidney Diseases (ARK) study. BMC Nephrol. 2020;21:20. doi:10.1186/s12882-020-1688-0

106 Currin SD, Gondwe MS, Mayindi NB, Chipungu S, Khoza BL, Tollman S, et al. Diagnostic accuracy of semiquantitative point of care urine albumin to creatinine ratio and urine dipstick analysis in a primary care resource limited setting in South Africa. BMC Nephrol. 2021;22:103. Medline:33743616 doi:10.1186/s12882-021-02290-5

107 Fabian J, Kalyesubula R, Mkandawire J, Hansen CH, Nitsch D, Musenge E, et al. Measurement of kidney function in Malawi, South Africa, and Uganda: A multicentre cohort study. Lancet Glob Health. 2022;10:e1159-e69. Medline:35839814 doi:10.1016/s2214-109x(22)00239-x

108 Naidoo S, Fabian J, Norris SA. Prevalence and associated risk factors for elevated blood pressure in young adults in South Africa. Cardiovasc J Afr. 2022;33:1-7. Medline:35976675 doi:10.5830/cvja-2022-036

109 Fabian J, Gondwe M, Mayindi N, Chipungu S, Khoza B, Gaylard P, et al. Chronic kidney disease (CKD) and associated risk in rural South Africa: A population-based cohort study. Wellcome Open Res. 2022;7:236. Medline:36457874 doi:10.12688/wellcomeopenres.18016.2

110 Gregson CL, Madanhire T, Rehman A, Ferrand RA, Cappola AR, Tollman S, et al. Osteoporosis, rather than sarcopenia, is the predominant musculoskeletal disease in a rural South African community where human immunodeficiency virus prevalence is high: A cross-sectional study. J Bone Miner Res. 2022;37:244-55. Medline:34694025 doi:10.1002/jbmr.4464

111 Currin S, Gondwe M, Mayindi N, Chipungu S, Khoza B, Khambule L, et al. Evaluating chronic kidney disease in rural South Africa: Comparing estimated glomerular filtration rate using point-of-care creatinine to iohexol measured GFR. Clin Chem Lab Med. 2021;59:1409-20. Medline:33711217 doi:10.1515/cclm-2020-1882

112 Kalyesubula R, Conroy AL, Calice-Silva V, Kumar V, Onu U, Batte A, et al. Screening for kidney disease in low- and middle-income countries. Semin Nephrol. 2022;42:151315. Medline:37001337 doi:10.1016/j.semnephrol.2023.151315

113 Simba H, Tromp G, Sewram V, Mathew CG, Chen WC, Kuivaniemi H. Esophageal cancer genomics in Africa: Recommendations for future research. Front Genet. 2022;13:864575. Medline:35401654 doi:10.3389/fgene.2022.864575

114 Abnet CC, Buckle GC, Chen Y, Dawsey SM, Kayamba V, Mwachiro MM, et al. Expanding oesophageal cancer research and care in Eastern Africa. Nat Rev Cancer. 2022;22:253-4. Medline:35246668 doi:10.1038/s41568-022-00458-1

115 Miyoshi J, Zhu Z, Luo A, Toden S, Zhou X, Izumi D, et al. A microRNA-based liquid biopsy signature for the early detection of esophageal squamous cell carcinoma: A retrospective, prospective and multicenter study. Mol Cancer. 2022;21:44. Medline:35148754 doi:10.1186/s12943-022-01507-x

116 Talukdar FR, Soares Lima SC, Khoueiry R, Laskar RS, Cuenin C, Sorroche BP, et al. Genome-wide DNA methylation profiling of esophageal squamous cell carcinoma from global high-incidence regions identifies crucial genes and potential cancer markers. Cancer Res. 2021;81:2612-24. Medline:33741694 doi:10.1158/0008-5472.Can-20-3445

117 Mwapagha LM, Chibanga V, Shipanga H, Parker MI. New insights from whole genome sequencing: BCLAF1 deletion as a structural variant that predisposes cells towards cellular transformation. Oncol Rep. 2021;46:229. Medline:34490482 doi:10.3892/or.2021.8180

118 Kandimalla R, Xu J, Link A, Matsuyama T, Yamamura K, Parker MI, et al. EpiPanGI Dx: A cell-free DNA methylation fingerprint for the early detection of gastrointestinal cancers. Clin Cancer Res. 2021;27:6135-44. Medline:34465601 doi:10.1158/1078-0432.Ccr-21-1982

119 van der Watt PJ, Okpara MO, Wishart A, Parker MI, Soares NC, Blackburn JM, et al. Nuclear transport proteins are secreted by cancer cells and identified as potential novel cancer biomarkers. Int J Cancer. 2022;150:347-61. Medline:34591985 doi:10.1002/ijc.33832

120 Ferndale L, Moodley M, Chen WC, Wadee R, Wright CA, Parker MI, et al. Processing and analysis of tissue samples from esophageal cancer patients in an African setting. Biopreserv Biobank. 2022;20:185-94. Medline:34388042 doi:10.1089/bio.2021.0030

121 Brown J, Stepien AJ, Willem P. Landscape of copy number aberrations in esophageal squamous cell carcinoma from a high endemic region of South Africa. BMC Cancer. 2020;20:281. Medline:32252688 doi:10.1186/s12885-020-06788-3

122 Matejcic M, Mathew CG, Parker MI. The relationship between environmental exposure and genetic architecture of the 2q33 locus with esophageal cancer in South Africa. Front Genet. 2019;10:406. Medline:31118947 doi:10.3389/fgene.2019.00406

123 Chen WC, Bye H, Matejcic M, Amar A, Govender D, Khew YW, et al. Association of genetic variants in CHEK2 with oesophageal squamous cell carcinoma in the South African Black population. Carcinogenesis. 2019;40:513-20. Medline:30753320 doi:10.1093/carcin/bgz026

124 Sammon AM, Ndebia EJ. Aetiology of oesophageal cancer in Africa - A review of historical and current evidence Glob J Health Sci. 2019;11:91-108.

125 Talukdar FR, di Pietro M, Secrier M, Moehler M, Goepfert K, Lima SSC, et al. Molecular landscape of esophageal cancer: Implications for early detection and personalized therapy. Ann N Y Acad Sci. 2018;1434:342-59. Medline:29917250 doi:10.1111/nyas.13876

126 Van Loon K, Mwachiro MM, Abnet CC, Akoko L, Assefa M, Burgert SL, et al. The African esophageal cancer consortium: A call to action. J Glob Oncol. 2018;4:1-9. Medline:30241229 doi:10.1200/jgo.17.00163

127 Dzobo K, Hassen N, Senthebane DA, Thomford NE, Rowe A, Shipanga H, et al. Chemoresistance to cancer treatment: Benzo-alpha-pyrene as friend or foe? Molecules. 2018;23:930. Medline:29673198 doi:10.3390/molecules23040930

128 Chen WC, Brandenburg J-T, Choudhury A, Hayat M, Sengupta D, Swiel Y, et al. Genome-wide association study of oesophageal cancer identifies trans-ethnic risk variants in African and Chinese populations. . Am J Hum Genetics. 2023 [in press]

129 Makubi A, Chillo P, Mutagaywa R, Balandya B, Kisenge P, Tarimo V, et al. Rationale, design and protocol of a cross-sectional study on pregnancy-related cardiovascular diseases in Tanzania (PRECARDT): Burden, characterisation and prognostic significance at delivery. BMJ Open. 2021;11:e049979. Medline:34972761 doi:10.1136/bmjopen-2021-049979

130 Masyuko SJ, Page ST, Polyak SJ, Kinuthia J, Osoti AO, Otieno FC, et al. Human immunodeficiency virus is associated with higher levels of systemic inflammation among Kenyan adults despite viral suppression. Clin Infect Dis. 2021;73:e2034-e42. Medline:33313687 doi:10.1093/cid/ciaa1650

131 Temu TM, Polyak SJ, Wanjalla CN, Mandela NA, Dabee S, Mogaka JN, et al. Latent tuberculosis is associated with heightened levels of pro-and anti-inflammatory cytokines among Kenyan men and women living with HIV on long-term antiretroviral therapy. AIDS. 2023;37:1065-75. Medline:36928263 doi:10.1097/qad.0000000000003523

132 Temu TM, Polyak SJ, Zifodya JS, Wanjalla CN, Koethe JR, Masyuko S, et al. Endothelial dysfunction is related to monocyte activation in antiretroviral-treated people with HIV and HIV-negative adults in Kenya. Open Forum Infect Dis. 2020;7:ofaa425. Medline:33094120 doi:10.1093/ofid/ofaa425

133 Temu TM, Wagoner J, Masyuko S, O'Connor A, Zifodya JS, Macharia P, et al. Central obesity is a contributor to systemic inflammation and monocyte activation in virally suppressed adults with chronic HIV in Kenya. AIDS. 2021;35:1723-31. Medline:34033591 doi:10.1097/qad.0000000000002956

134 Temu TM, Zifodya JS, Polyak SJ, Wagoner J, Wanjalla CN, Masyuko S, et al. Antiretroviral therapy reduces but does not normalize immune and vascular inflammatory markers in adults with chronic HIV infection in Kenya. AIDS. 2021;35:45-51. Medline:33055570 doi:10.1097/QAD.0000000000002729

135 Kayongo A, Bartolomaeus TUP, Birkner T, Marko L, Lober U, Kigozi E, et al. Sputum microbiome and chronic obstructive pulmonary disease in a rural Ugandan cohort of well-controlled HIV infection. Microbiol Spectr. 2023;11:e0213921. Medline:36790203 doi:10.1128/spectrum.02139-21

136 Kayongo A, Robertson NM, Siddharthan T, Ntayi ML, Ndawula JC, Sande OJ, et al. Airway microbiome-immune crosstalk in chronic obstructive pulmonary disease. Front Immunol. 2022;13:1085551. Medline:36741369 doi:10.3389/fimmu.2022.1085551

137 Kayongo A, Wosu AC, Naz T, Nassali F, Kalyesubula R, Kirenga B, et al. Chronic obstructive pulmonary disease prevalence and associated factors in a setting of well-controlled HIV, A cross-sectional study. COPD. 2020;17:297-305. Medline:32462945 doi:10.1080/15412555.2020.1769583

138 Hassan MO, Duarte R, Mabayoje VO, Dickens C, Lasisi AO, Naicker S. Design and methods of the prevalence and pharmacogenomics of tenofovir nephrotoxicity in HIV-positive adults in south-western Nigeria study. BMC Nephrol. 2020;21:436. Medline:33066744 doi:10.1186/s12882-020-02082-3

139 Mashili F, Joachim A, Aboud S, McHembe M, Chiwanga F, Addo J, et al. Prospective exploration of the effect of adiposity and associated microbial factors on healing and progression of diabetic foot ulcers in Tanzania: Study protocol of a longitudinal cohort study. BMJ Open. 2019;9:e031896. Medline:31848165 doi:10.1136/bmjopen-2019-03189
